# Supplementary figures and images for: Transcriptomic study of WSSV infection in Litopenaeus vannamei lymphoid organ via single nuclei RNA sequencing
Source: PLoS One. 2026 Apr 27;21(4):e0348006. doi: 10.1371/journal.pone.0348006 (PMC13119866; doi:10.1371/journal.pone.0348006)

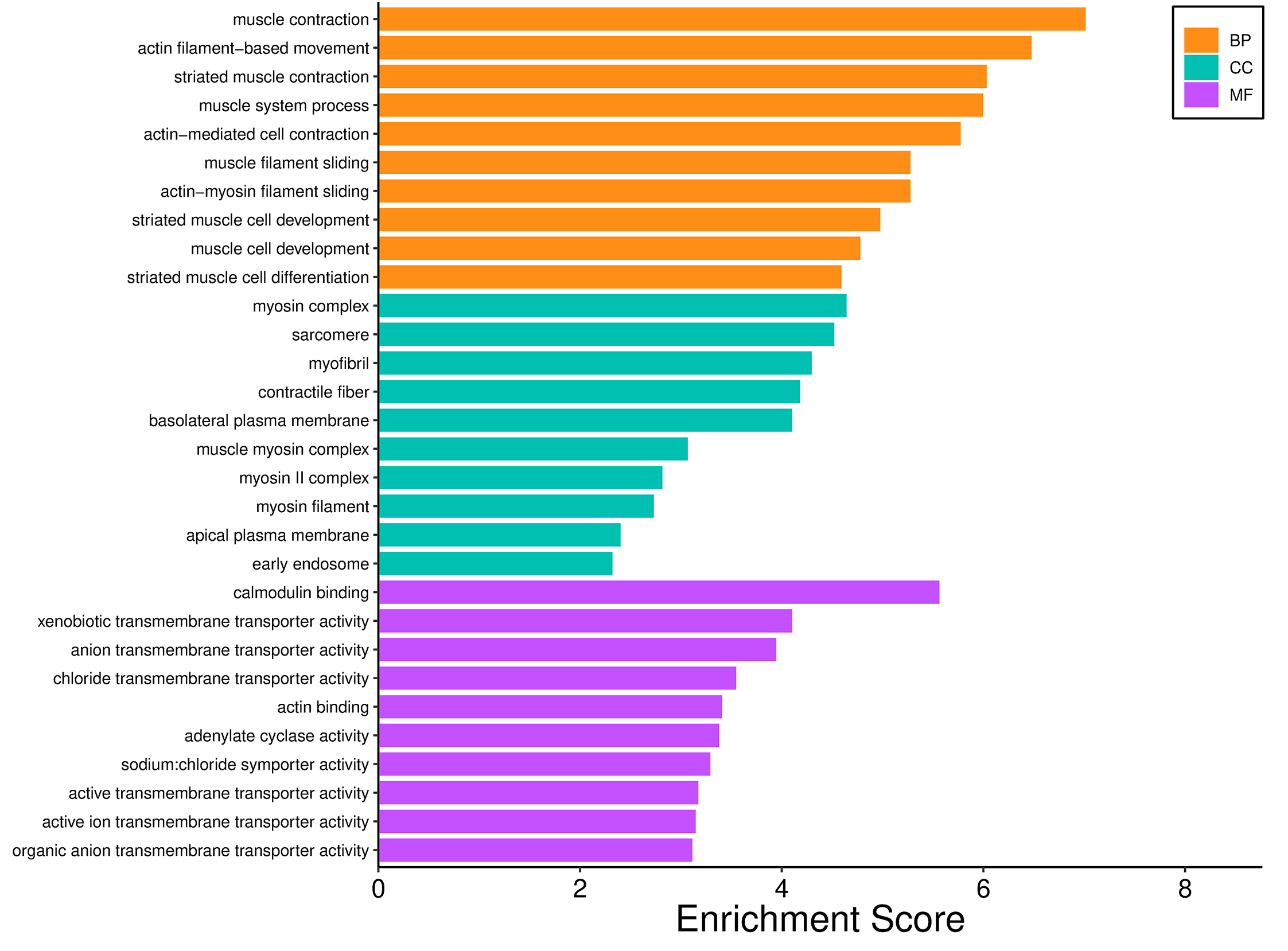

Supplement: S1 Fig — The figure displays the top 10 biological processes (BP), top 10 cellular components (CC) and top 10 molecular functions (MF) enriched in the lymphoid organ during WSSV infection based on GE genes. The length of the bar corresponds to the enrichment, calculated as -log10 of the p-value. (TIF) [file pone.0348006.s002.tif]

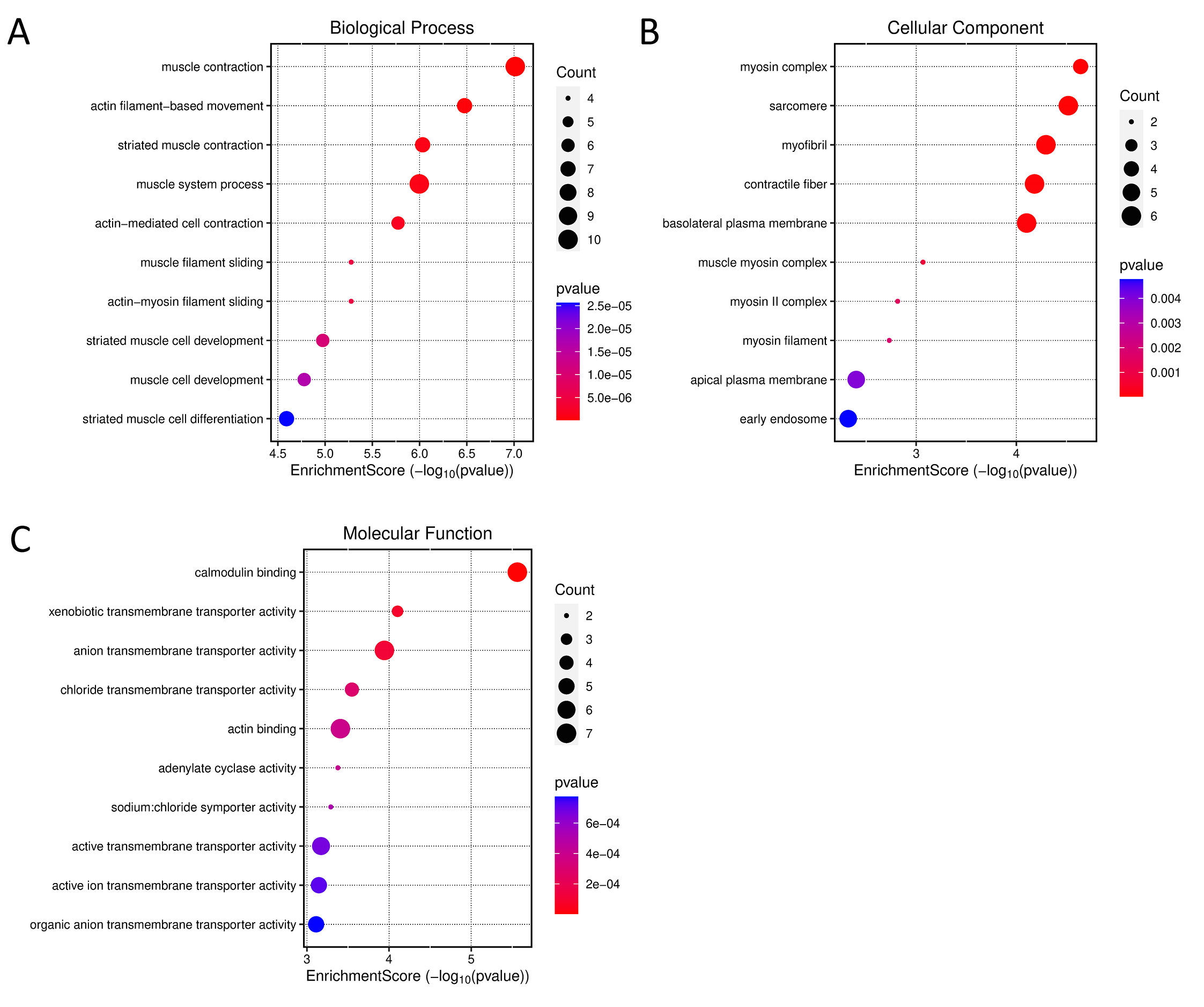

Supplement: S2 Fig — The figure displays the top 10 biological processes (A), top 10 cellular components (B) and top 10 molecular functions (C) enriched in the lymphoid organ during WSSV infection based on GE genes. The “count” displays the number of DE genes that contributed to the category. The “p-value” shows the significance where p < 0.05 is significant. The enrichment score is displayed on the x-axis. (TIF) [file pone.0348006.s003.tif]

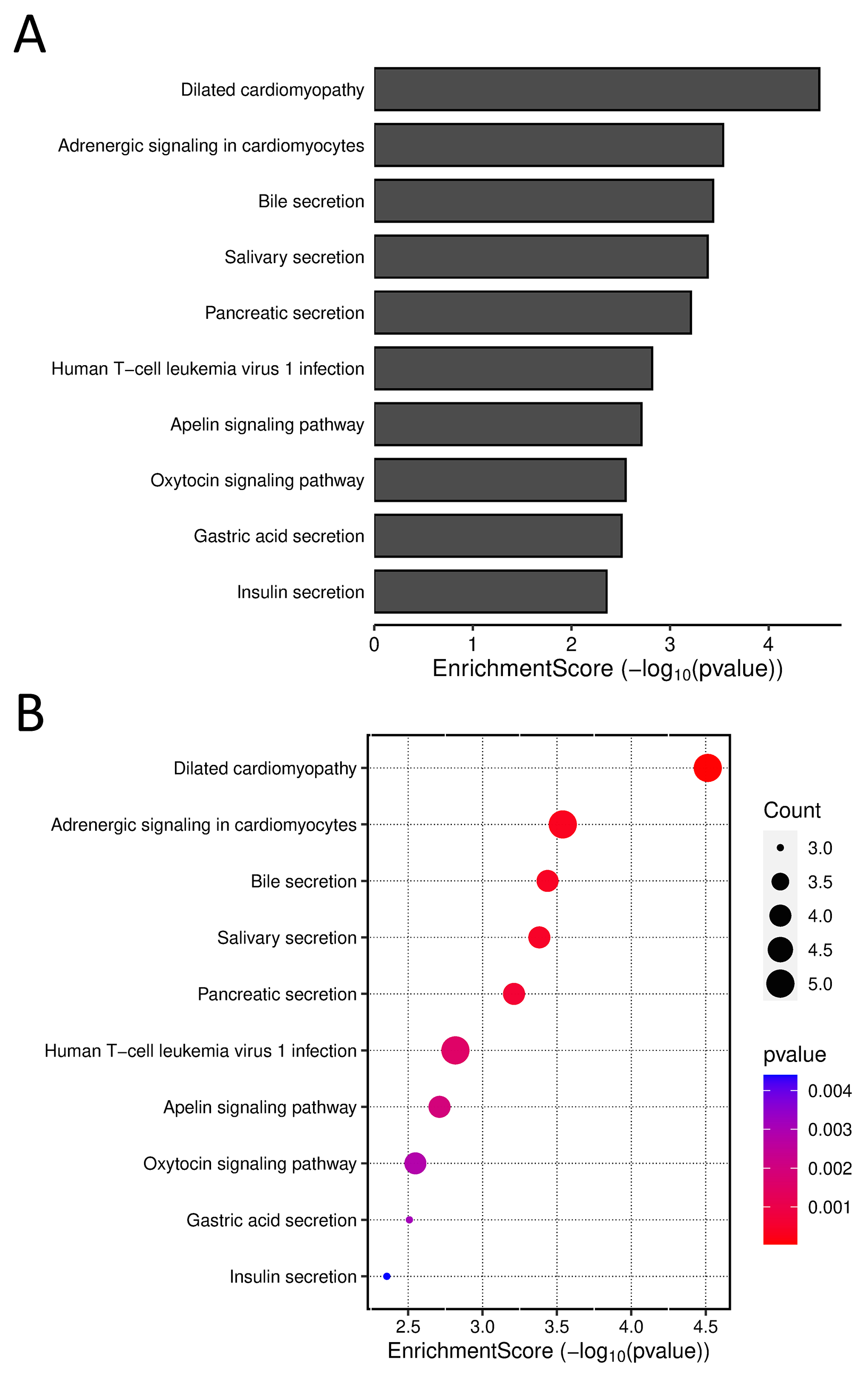

Supplement: S3 Fig — The figures represent the top 10 KEGG pathways significantly enriched in lymphoid organ tissue during WSSV infection based on DE genes. The “count” displays the number of DE genes that contributed to the category. The “p-value” shows the significance where p < 0.05 is significant. The enrichment score is displayed on the x-axis, calculated as -log10 of the p-value. The data is displayed as Barplot (A) and a Dotplot (B). (TIF) [file pone.0348006.s004.tif]
